# An Optimal Design Method for Lightweight Heating Film of Anisotropic Heat Conduction Substrate Based on Surrogate Model

**DOI:** 10.3390/mi15080970

**Published:** 2024-07-29

**Authors:** Zheng Deng, Qingkui Yu, Jingyu Liu, Yanan Wang, Shoubing Yan, Nana Huai, Jingze Zhang, Huaxing Gao

**Affiliations:** China Aerospace Components Engineering Center, No. 104 Youyi Rd. Haidian District, Beijing 100048, China; bjingdz@163.com (Z.D.); jingyu_xx@hotmail.com (J.L.); wyn.wgn@163.com (Y.W.); bingbinghappy521@163.com (S.Y.); huainana1994@163.com (N.H.); ureyzjz@163.com (J.Z.); ghx.678@163.com (H.G.)

**Keywords:** PSO-BP surrogate model, NSGA-II, lightweight heating film, multi-objective optimization

## Abstract

In space missions, heating films are crucial for uniformly heating onboard equipment for precise temperature control. This study develops an optimization method using surrogate models for lightweight anisotropic substrate thermal conductive heating films, meeting the requirements of uniform heating in thermal control for space applications. A feedforward neural network optimized by particle swarm optimization (PSO) was employed to create a surrogate model, mapping design parameters to the temperature uniformity of the heating film. This model served as the basis for applying the NSGA-II algorithm to quickly optimize both temperature uniformity and lightweight characteristics. In this study, the PSO-BP surrogate model was trained using heating film thermal simulation data, and the surrogate model demonstrated an accurate prediction of the mean square error (MSE) of the predicted temperature difference within 0.0168 s. The maximum temperature difference in the optimal model is 1.188 ℃, which is 30.5 times lower than before optimization, and the equivalent density is only increased by 3.9%. In summary, this optimization design method effectively captures the relationships among various parameters and optimization objectives. Its superior computational accuracy and design efficiency offer significant advantages in the design of devices such as heating films.

## 1. Introduction

Heating film is a thin-film device that uses the resistive thermal effect of regularly distributed heating wires to uniformly heat complex curved surfaces [1]. Due to its ease of use and high heating efficiency, it is widely used in the temperature control and heating of aerospace devices [2,3,4,5]. As space missions become increasingly complex and diverse [6,7], existing heating films based on low thermal conductivity materials like PI and PDMS are unable to meet the current mission requirements due to uneven heating [2,8,9]. An effective approach to enhance the uniformity of heat distribution is to increase the density of the internal heating wires [10]. However, this results in a notable increase in the mass of the heating film, which is a drawback in the context of weight reduction in spacecraft [11,12]. It is evident, therefore, that the simultaneous fulfilment of the heating uniformity and lightweight requirements for spacecraft thermal control heating films necessitates the implementation of a meticulous material and structural design.

At present, researchers are integrating heat conduction theory and material design to investigate the heating films, with the objective of enhancing their in-plane heating uniformity. Zhao, Z. et al. [13] used anisotropic materials as the heating film substrate, designing the thermal conductivity of the material to achieve uniform in-plane heating by increasing the in-plane thermal conductivity. The aforementioned design enables directional control of heat flow within the heating film. However, the incorporation of high-density thermal conductive materials results in an increase in material density [14,15]. The combination of heating film materials with structural design represents a more effective approach. Lee, S.M. et al. [16] employed a colloidal deposition method to grow silver metal grids on the surface of the substrate material, achieving a maximum heating temperature of 245 °C at an input voltage of 7 V. This method also resulted in high-efficiency electric heating and light transmittance. Han, D. et al. [17] designed a sandwich-structured transparent conductive heating film, embedding silver nanowires between two substrate layers. This resulted in excellent mechanical deformation performance and high electrothermal conversion efficiency. The preparation of high-performance functional materials and the rationale of structural designs can significantly enhance the thermal performance of heating films. Nevertheless, this frequently results in a trade-off among other properties, such as lightweighting, transparency, and stretchability. Balancing the various properties of materials to meet specific application requirements represents a significant challenge.

In recent years, the field of artificial intelligence has witnessed a remarkable advancement, with significant potential in the domain of device optimisation design [18,19,20]. Researchers have leveraged the powerful nonlinear relationship fitting capabilities of neural networks to facilitate device design through the establishment of surrogate models [21]. Xiong, Y. et al. [22] developed a surrogate model for the thermal design of spacecraft payloads under on-orbit flight conditions, based on deep neural networks. This model has accurately predicted the temperatures at key points of a space telescope under different material parameters. This achievement represents a significant step towards the assisted design of the device. When the objectives of the optimisation process are clearly defined, integrating the design of devices with artificial intelligence technology represents an effective approach. Ayman Negm et al. [23] applied deep learning methods to the design of intelligent cooling metasurfaces for spacecraft. They employed convolutional neural networks to construct tunable and reconfigurable metasurface structures, combining them with a pattern search method for optimization. This approach resulted in a 28% reduction in the coating thickness of the optimized vanadium dioxide metasurface without compromising performance. However, the iterative nature of intelligent algorithms requires substantial computation, leading to lower optimization efficiency and posing challenges for practical application.

Machine learning methods have shown initial success in assisting device design, but there are still some deficiencies and challenges in this field [24]. Currently, the functional integration of devices and the environments they face are becoming increasingly complex, requiring devices to simultaneously meet multiple design objectives. Therefore, relying solely on machine learning methods to establish surrogate models for the empirical design of devices no longer meets current demands. To address the contradiction between achieving uniform heating and lightweight design for spacecraft thermal control heating films, this study proposes a multi-objective optimization design method for anisotropic thermal conductivity heating films based on a PSO-BP surrogate model combined with the NSGA-II algorithm. This method uses a feedforward neural network to predict the temperature distribution of the heating film under different design parameters and employs a genetic algorithm to solve for the true Pareto front solutions. The predictions of the surrogate model and the optimization results are thoroughly discussed and analysed. Compared to the original heating film, the optimized structure shows outstanding performance: the temperature difference is reduced by 35.1 °C with only a 3.9% increase in equivalent density. This method, based on machine learning combined with various intelligent optimization algorithms, addresses the optimal design problem for aerospace devices and aims to further integrate device design with the field of artificial intelligence.

## 2. Research Object

**Figure 1:**
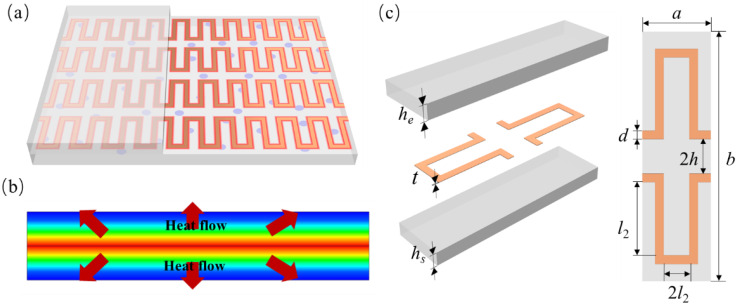
Research object. (**a**) Schematic diagram of the uniformly heating film with serpentine heating wires embedded in anisotropic thermally conductive polymer layers; (**b**) schematic diagram of heat flow uniformity within the anisotropic substrate heating film; (**c**) heating film unit cell model.

The necessity for lightweight designs in order to meet the demanding requirements of aerospace missions regarding payload, energy consumption, and flight performance is driving the continuous evolution of onboard equipment [25]. At the same time, it is essential to ensure that the necessary performance is maintained. Addressing the specific need for high heating uniformity in surface heating films for certain aerospace equipment, this paper innovatively proposes a design for a uniformly heating film using an anisotropic substrate. As shown in Figure 1a,c, the heating film consists of substrate materials and a serpentine heat source. The top and bottom layers of the substrate material are polydimethylsiloxane (PDMS) doped with oriented boron nitride (BN), which significantly enhances the thermal conductivity in the horizontal direction, thereby achieving an ideal heat flow distribution (shown in Figure 1b). To design the heating film effectively, this study employed a square wave design for the arrangement of heating wires to enhance the heating density and customization potential of the film. By integrating an equivalent thermal model of the heating film for simulation analysis, this research extensively examined the relationship among factors such as the oriented doping filler ratio, material of the metal heating wires, and size parameters of the heating wires with the in-plane temperature uniformity and equivalent density of the heating film based on thermal simulation results.

## 3. Research Contents

### 3.1. PSO-BP Surrogate Model

PSO-BP is a backpropagation (BP) neural network model optimised via particle swarm optimization (PSO) [26,27]. PSO is a population-based optimization algorithm that simulates the foraging behaviour of bird flocks, seeking the optimal solution through the movement and interaction of particles within the search space [28,29]. The BP neural network is a multi-layer feedforward neural network based on the error backpropagation algorithm, comprising an input layer, an output layer, and one or more hidden layers [30,31]. The network’s weights and thresholds are adjusted by calculating the error gradient in order to minimise the discrepancy between the network output and the desired output.

**Figure 2:**
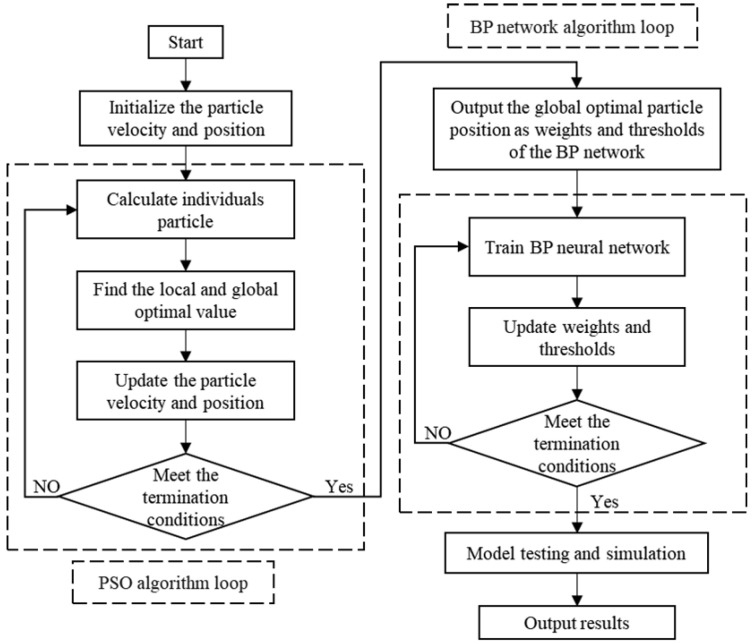
PSO-BP neural network model training diagram.

In the PSO-BP model, each particle represents a set of weights and thresholds for the neural network. The position and velocity of the particles correspond to the adjustments of these weights and thresholds. The error gradient calculated using the BP algorithm guides the particles’ movement direction and speed updates. The PSO-BP model combines the parallelism and global search capability of PSO with the adaptability of the BP neural network, accelerating the training process and overcoming the BP algorithm’s tendency to become stuck in local optima, thereby improving the prediction accuracy of the BP neural network. The calculation process of the PSO-BP model is shown in Figure 2, and the detailed implementation process includes the following steps:

1. Initialize neural network: Define the structure of the neural network and initialize the weights and thresholds. Initialize the positions and velocities of the particle swarm.
2. Fitness calculation: Compute the fitness value of each particle using the BP neural network.
3. Identify best solutions: Identify the personal best (pBest) for each particle and the global best (gBest) among all particles based on their fitness values.
4. Update velocity and position: Update the velocity and position of each particle based on the pBest, gBest, current position, and velocity.
5. Iterative optimization: Repeat steps 2–4, iterating until the maximum number of iterations is reached, fitness values stabilize, or other stopping criteria are met.
6. Assign optimal weights and thresholds: Assign the weights and thresholds from the global best solution found by the PSO algorithm to the BP neural network.
7. Train BP neural network: Train the BP neural network through forward propagation and error backpropagation until maximum iterations are reached, the error falls below a threshold, or other stopping criteria are met.
8. Output final model: Output the trained neural network as the final model.

### 3.2. Multi-Objective Optimization Algorithm

**Figure 3:**
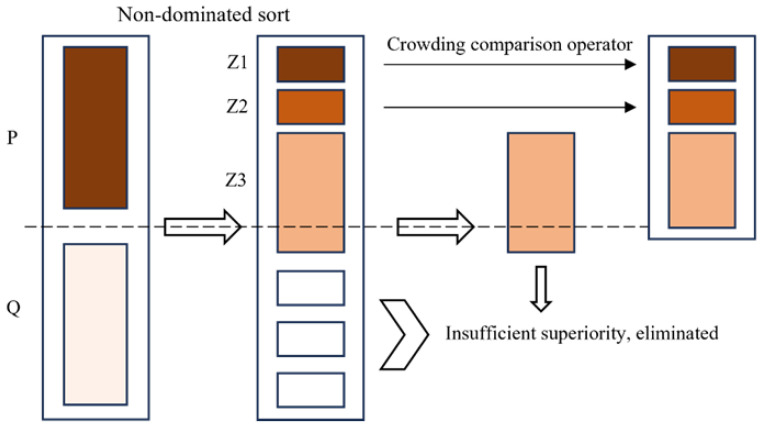
Diagram of NSGA-II algorithm.

The solving of multi-objective optimization problems primarily employs two distinct methodologies: traditional methods and intelligent optimization algorithms [32,33]. Traditional methods, such as weighted sum and constraint methods, convert multi-objective functions into a single objective function and then seek the extreme values of this function. Nevertheless, the parameter settings inherent to these methods can potentially complicate the problem-solving process. The non-dominated sorting genetic algorithm (NSGA) is an intelligent optimization algorithm designed to solve multi-objective problems [34]. The algorithm’s principal innovation is the non-dominated sorting process, which categorises individuals in the population into distinct levels, each comprising a set of non-dominated solutions. The NSGA algorithm prioritises individuals with higher non-domination ranks during the selection process, thereby maintaining the diversity of solutions in the population. NSGA-II, an improved version of NSGA, was proposed by Kalyanmoy Deb in 2000 [35,36]. In comparison to NSGA, NSGA-II introduces a fast non-dominated sorting algorithm, crowding distance and crowding comparison operators, and an elitist strategy, significantly enhancing the algorithm’s performance and efficiency. The principles of the algorithm are illustrated in Figure 3. Among intelligent optimization algorithms, NSGA-II has unique advantages over many similar algorithms such as multiple-objective GA (MOGA), multiple-objective PSO (MOPSO), and multiple-objective GWO (MOGWO). It has demonstrated unique advantages and excellent performance in multiple small-scale simple problem optimization tasks [37,38,39] and can achieve the optimal solution selection under limited computing resources. Since the structure of the heating film optimization problem studied in this paper is relatively simple, the NSGA-II algorithm combined with the PSO-BP agent model can achieve a fast solution to this multi-objective optimization problem. The implementation principle is as follows:

1. Define objective functions and constraints: Define the optimization objectives and constraints of the problem, which limit the range of feasible solutions.
2. Initialize population: Generate an initial population of solutions randomly to serve as the starting point for the algorithm.
3. Evaluate fitness: Use the PSO-BP surrogate model to calculate the fitness of each solution in the initial population.
4. Select parents: Use non-dominated sorting and crowding distance to select the better solutions as parents for the next generation.
5. Crossover and mutation: Apply crossover and mutation operations to the parent solutions to produce offspring. Crossover simulates genetic recombination, while mutation introduces genetic variations.
6. Non-dominated sorting and crowding distance calculation: Perform non-dominated sorting to determine the positions of new solutions on the Pareto front and calculate crowding distances to evaluate solution diversity.
7. Iterative optimization: Repeat steps 4–6 to iteratively optimize the solution set until the stopping criteria are met, such as reaching the maximum number of generations or achieving a stable fitness value.
8. Output Pareto optimal front set: Output the Pareto optimal front set of solutions obtained through the optimization process.

## 4. Experimental Content

**Figure 4:**
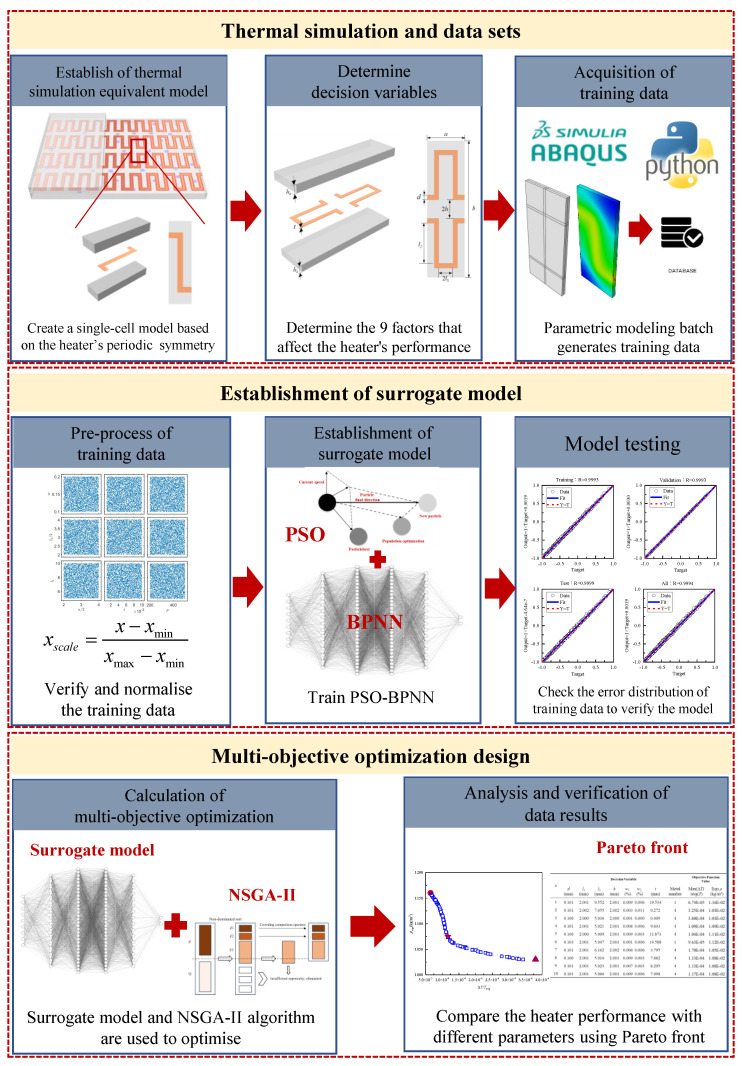
Experimental technology roadmap.

The heating film was designed and studied by combining the PSO-BP neural network as a surrogate model with the NSGA-II algorithm. The experimental technical route is depicted in Figure 4. Firstly, the surrogate model is driven by thermal simulation data, and the training data set is generated in batches by establishing an equivalent thermal simulation model and parameterised modelling. Secondly, the training data are normalised, and a BP neural network surrogate model is established. The initial weights and thresholds of the BP neural network are optimised in conjunction with the PSO algorithm in order to facilitate the training process. Following multiple rounds of training, the performance of the surrogate model is verified from multiple angles. Finally, the relationship between the parameters and the performance of the heating film established by the surrogate model is utilised to optimise the temperature uniformity and equivalent density within the heating film surface via the NSGA-II algorithm. The calculated Pareto front set is subsequently subjected to discussion and analysis.

### 4.1. Training Data Acquisition

**Figure 5:**
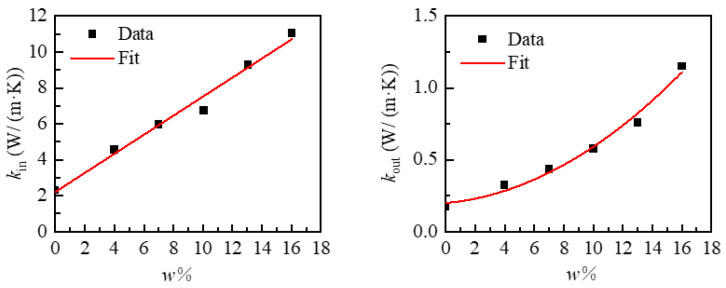
Relationship of BN mass percentage *w* and thermal conductivity *k* in PDMS-based anisotropic thermal conductivity materials.

The subject of this study was the anisotropic substrate thermal conductive heating film, which is a periodic structure comprising an upper substrate, a lower substrate, and an intermediate periodic heating wire. Heating wires in the heating film can generally be made of conductive metals such as gold, silver, copper, and aluminium, chosen according to specific usage requirements. This study did not have specific requirements for material conductivity and heating efficiency; therefore, the heating wires were selected from the four materials listed in Table 1, as shown. In order to achieve directional regulation of the heating film heat flow, the PDMS-based anisotropic thermal conductivity material, designed by Hong, H. et al. [40], was selected as the substrate. Figure 5 depicts the BN filling rate in the material and the thermal conductivity of the material in all directions. The distribution was then subjected to a fitting procedure, resulting in the following relationship:

(1) $$\begin{array}{c} k_{in}=2.214+0.531w,\\ k_{out}=0.199+0.01w+0.003w^{2},\\ \rho =\left[2290\times w+1030\times \left(100-w\right)\right]\times 0.01, \end{array}$$

where *k_in_* represents the thermal conductivity within the material surface, while *k_out_* denotes the thermal conductivity outside the material surface. The parameter *w* signifies the filling mass percentage of BN in the material, while *ρ* represents the equivalent density of the material.

**Figure 6:**
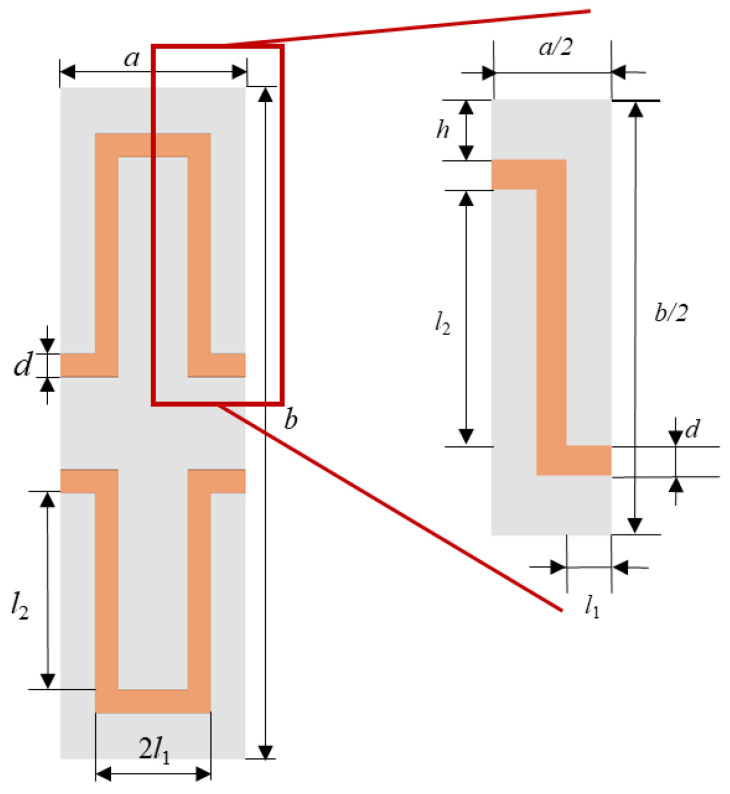
Unit cell and one-fourth unit cell of heater film.

This paper selects the one-fourth unit cell heater structure depicted in Figure 6 as the thermal analysis model, as it is representative of the periodic and symmetrical structural characteristics of the heating film. The structural design and material selection of the heating film have a direct impact on the uniformity of the heating and the thermal efficiency of the system. This encompasses the structural parameters of the heating film, including the heating wire width *d*, one-fourth unit cell transverse heating wire length *l*_1_, one-fourth unit cell longitudinal heating wire length *l*_2_, longitudinal heating wire spacing *h*, heating wire thickness *t*, and the heating film material parameters: lower substrate BN filling rate *w*_1_, upper substrate BN filling rate *w*_2_, metal wire material (thermal conductivity *k*, density *ρ*), etc. In accordance with the aforementioned methodology, this work selected nine parameters, as shown in Table 2, as variables and calculated the thermal simulation results of one-fourth of the unit cell within the constraints of the variables.

**Figure 7:**
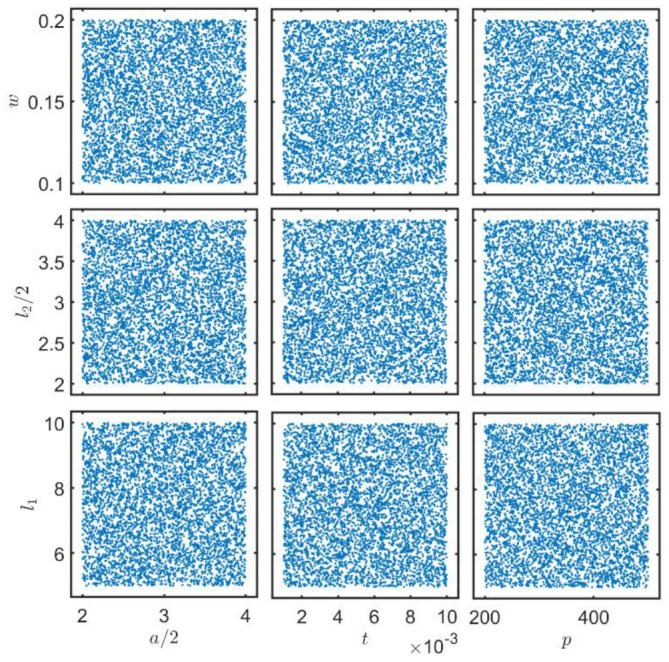
Spatial distribution of LHS sampling parameters.

Appropriate training data are crucial for enabling a neural network model to effectively learn the intrinsic physical knowledge embedded in the data. To achieve uniformity in experimental sampling, this study employed the Latin hypercube sampling (LHS) method for the experimental design, generating a data set of 5000 samples with nine input parameters. LHS represents a space-filling technology that can be employed to obtain more optimal initial sample points by utilizing fewer points. This is achieved by dividing the sampling space into multiple intervals with the same probability and then sampling in each interval. In order to verify the rationality of the sampling space, the sampling space of the nine parameters in the input parameters was plotted, as shown in Figure 7. The uniform distribution of the data space indicates that the input parameters comprehensively covered the entire sample space.

In order to facilitate the generation of training data sets for training surrogate models, this work employed parametric modelling and established a heat transfer model of heating films with varying sizes and material properties in Abaqus 2023. A total of 5000 sets of data were calculated by combining the input parameter data set with the Python batch processing function. Firstly, in the thermal simulation calculation, the heat transfer coefficient of the upper and lower surfaces of the heating film was 10 W/m^2^·K. In order to facilitate a comparison of the performance of the heating film and the actual working conditions, the heating film was subjected to a heat load of 0.1 W, with the seeds arranged at a fixed interval of 0.05 mm to generate a heat transfer-type hexahedral network. Secondly, we read 5000 sets of input data sets generated by MATLAB 2023 using Python 3.9. Each set of data was incorporated into the parametric modelling script for modelling purposes, and the simulation calculation was submitted in batches via Windows batch scripts. Finally, Python 3.9 was employed to read the file in order to extract the batch data of .odb files generated by 5000 simulations. The requisite data, including the maximum temperature difference and the average temperature of the bottom temperature of the heating film, were obtained, thereby forming a temperature data set comprising 5000 sets of heating film thermal simulation calculations.

### 4.2. Training Method

In the training data set for the neural network, discrepancies in the physical significance of various parameters may result in discrepancies in data scales and value ranges; such issues may have a detrimental impact on the final training results of the model. Consequently, it is essential to normalise the data in order to mitigate the impact of differing data scales and to enhance data precision and comparability. Two common normalization methods are min-max normalization and Z-score normalization. Min-max normalization is a linear transformation of the original data, which effectively eliminates the influence of differing data scales among samples while preserving the characteristics of the data distribution. This work employed the min-max normalization method to standardize all parameters in the training data set to the range of −1 to 1. Based on the findings of grid training, 80% of the data were selected from the normalized data set for network training, while 10% of the data were utilized for network testing and 10% of the data were utilized for network validation.

**Figure 8:**
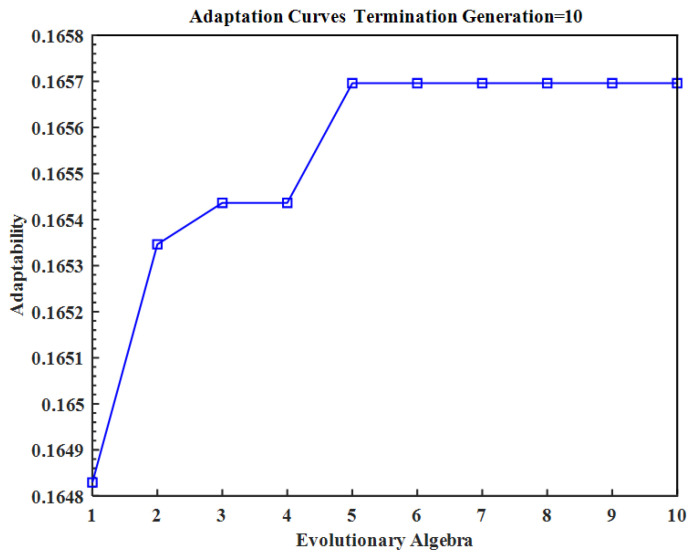
PSO iterative evolution process.

This work developed and designed the heating film neural network surrogate model based on MATLAB R2023b software. This model comprised two distinct modules: an initial optimization module and a BP neural network module. The initial optimization module was implemented through the use of PSO, and the population size and iteration number were selected in a manner that was consistent with the structure of the BP neural network. A comparison of the results obtained for different values indicated that the population size selected in this paper, namely, 20, and the iteration number, namely, 10, had the most beneficial effect on the BP neural network optimization. Figure 8 depicts the evolution curve with the inverse of the loss function of the BP neural network serving as the fitness function. In the BP neural network component, nine design parameters were employed as inputs, and the maximum temperature difference Δ*T* at the bottom of the heating film and the average temperature *T_avg_* at the bottom were utilized as outputs to construct a neural network model. The network structure was configured as 9-16-32-32-16-2, and the ADAM optimizer was employed for training. The initial learning rate was 0.01, the learning decay rate was 0.05, the number of epochs was 100, and the minimum batch size was 512. The neural network training was conducted on GPU: NVIDIA GeForce GTX 1650, purchased from NVIDIA (Santa Clara, CA, USA) manufacturers on Taobao platform. The training process was terminated when the MSE reached a value of 2 × 10^−4^.

### 4.3. Model Performance Evaluation

After the model training was completed, it was necessary to evaluate the model’s performance by determining its accuracy and computational speed. This assessment ensured the optimization process proceeded smoothly and effectively. The assessment of model efficacy frequently employs a number of indicators, including the mean square error (MSE), the correlation coefficient (R), and other relevant metrics. These indicators help in evaluating the accuracy and reliability of the model, ensuring it meets the required performance standards. MSE is employed to assess the discrepancy between the model prediction value and the actual observation value. A smaller MSE value indicates a greater degree of model fit. The determination coefficient is a measure of the goodness of fit of the model. The error of each output parameter can be employed to assess the efficacy of the model.

**Figure 9:**
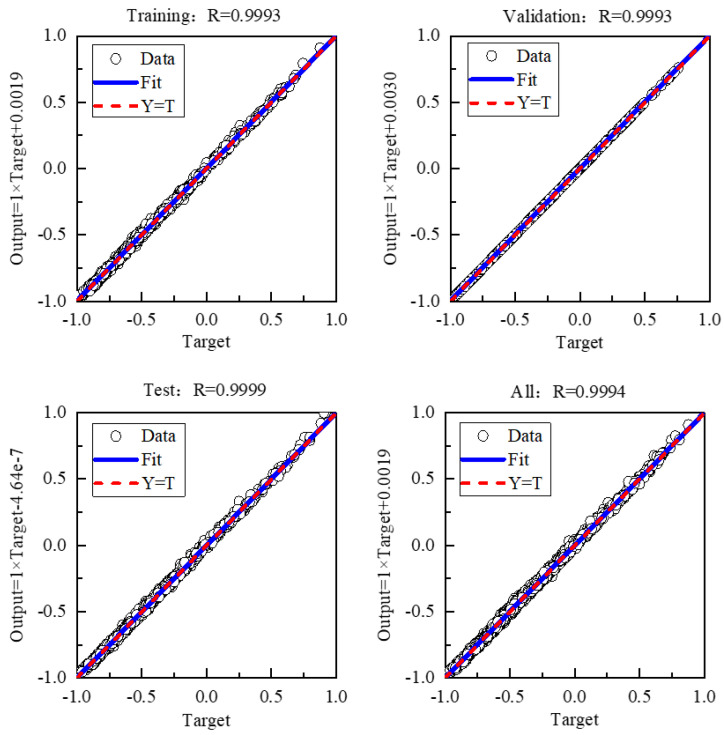
Regression of the surrogate model in the source domain.

In order to evaluate the prediction performance of the constructed PSO-BP neural network model, this work studied the results of the mean square error and correlation coefficient of the surrogate model for the prediction results. As illustrated in Figure 9, the mean square error (MSE) of the validation set of the model was (0.0373, 0.2492) °C, with an R value of 0.9993. The MSE of the test set was (0.0412, 0.2517) °C, with an R value of 0.9999. The MSE of the prediction model was exceedingly minimal. The regression analysis of the model indicated that the R-value for the training set, validation set, and test set was greater than 0.999. The fitting effects of each data set were essentially identical, indicating that the model did not exhibit overfitting or underfitting issues and was suitable for practical applications.

**Figure 10:**
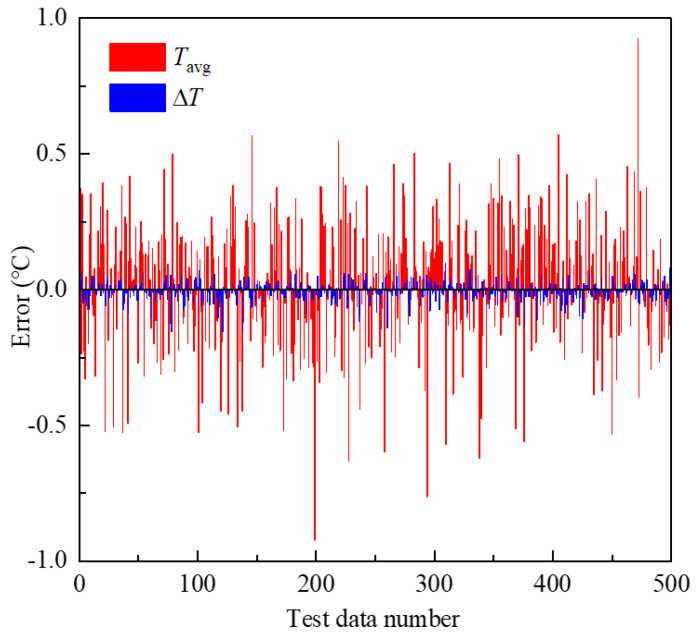
The error of the output parameters from the test set.

A further statistical analysis of Δ*T* and *T_avg_* is presented in Figure 10. In the test data set, the absolute value of the error in Δ*T* was less than 0.1 °C, and the absolute value of the error in *T_avg_* was less than 1 °C. Due to the fact that the two output parameters differed by an order of magnitude, the surrogate model’s predictive performance was comparable for both parameters. Additionally, considering that the maximum prediction error was less than 5% of the output results, the relatively small error was acceptable for the optimization calculations.

### 4.4. Optimization Design Method

The surrogate model can swiftly and accurately compute the temperature distribution for different heating film structures and material designs. Combined with intelligent optimization algorithms, it enables rapid iterations to achieve the optimal design. The present study employed a multivariable approach to identify the optimal configuration of a heating film. Nine variables, as detailed in Table 2, were selected as decision variables, with the objective of minimizing the $\rho_{avg}$ value of equivalent density at the minimum temperature difference Δ*T* of the bottom surface of the heating film. In order to enhance the heating efficiency by ensuring the uniform heating of the heating film, the maximum temperature difference/average temperature squared ($\Delta T\left(X\right)/T_{avg}^{2}\left(X\right)$) on the bottom surface of the heating film was identified as one objective function. The $\rho_{avg}$ of the heating film was then employed as another objective function, with the objective of achieving lightweighting of the heating film. In consideration of the manufacturing process and application background of the heating film, the optimization range of the structural and material properties of the heating film was constrained in this study. The mathematical expression for this optimization problem is as follows:

(2) $$\begin{array}{c} \mathrm{Find}:\;X=d,l_{1},l_{2},h,w_{1},w_{2},k,\rho ,\\ \mathrm{Minimize}:\;F\left(X\right)=\frac{\Delta T\left(X\right)}{T_{avg}^{2}\left(X\right)},\frac{M\left(X\right)}{V\left(d,l_{1},l_{2},h\right)},\\ s.t.:0.1\leq d\leq 0.2,\\ 2\leq l_{1}\leq 4,\\ 5\leq l_{2}\leq 10,\\ 2\leq h\leq 4,\\ 0.001\leq t\leq 0.01,\\ 0\leq w_{1}\leq 20,\\ 0\leq w_{2}\leq 20,\\ 200\leq k\leq 500,\\ 2\times 10^{3}\leq \rho \leq 20\times 10^{3}, \end{array}$$

**Figure 11:**
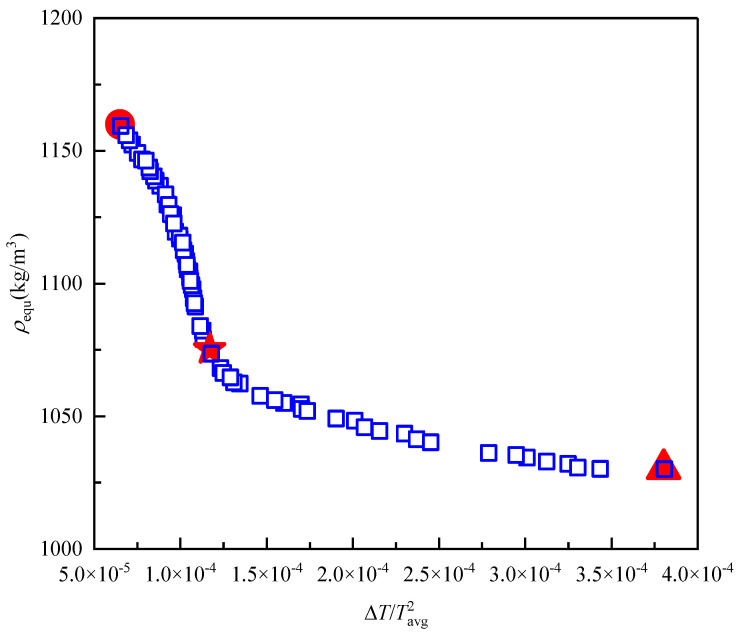
The distribution of Pareto front solutions calculated by NSGA-II algorithm.

In light of the multi-objective optimization problem elucidated in the preceding formula, this work employed the NSGA-II algorithm to address it. Following the completion of the iteration process, a total of 70 groups of Pareto front solutions were obtained, as illustrated in Table 3. The corresponding Pareto optimal front set diagram is presented in Figure 11. As illustrated in Figure 11, there was a negative correlation between the objective function $\Delta T\left(X\right)/T_{avg}^{2}\left(X\right)$ and the heating film equivalent density ($\rho_{avg}$).

## 5. Results and Discussion

Intelligent optimization algorithms effectively address the challenges of multi-objective optimization, providing a wider range of solution options for different optimization goals. As shown in Figure 11, the NSGA-II optimization algorithm yields a set of Pareto front solutions. To further investigate the trade-offs among these solutions, this study discusses the selection of heating film design schemes based on the optimization objectives.

**Figure 12:**
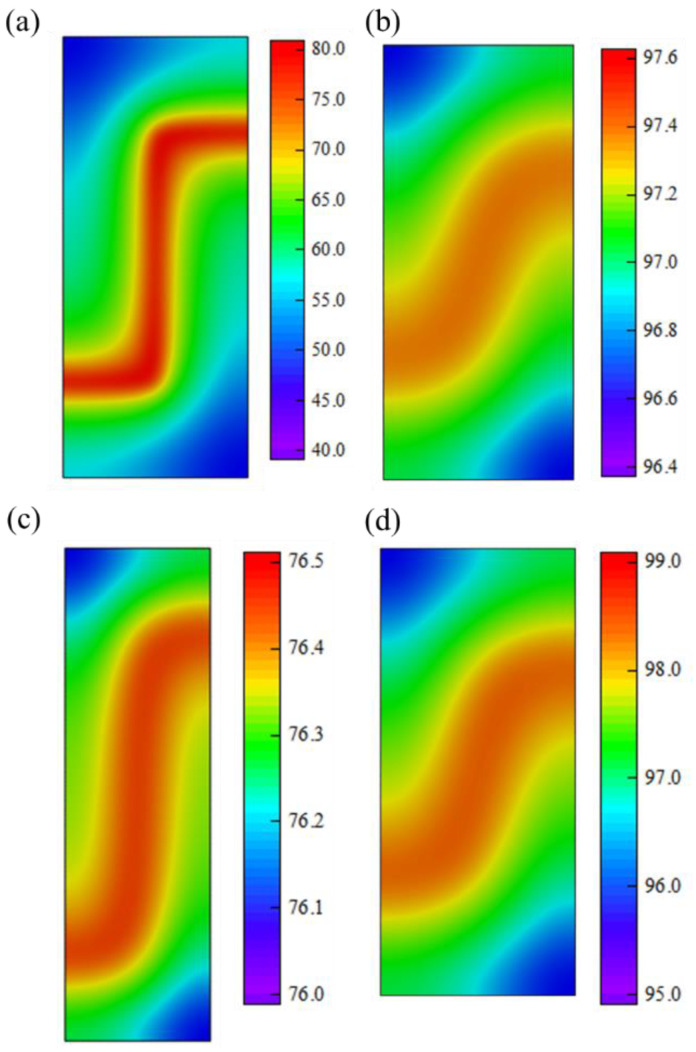
Temperature field on the bottom surface of heating film one-fourth unit cell. (**a**) Original design before optimization. (**b**) An optimal design selected from Pareto frontiers. (**c**) Model #47 in Pareto frontier. (**d**) Model #3 in Pareto frontier.

This study utilizes an improved genetic algorithm combined with the PSO-BP neural network surrogate model to compute 70 sets of optimal solutions, as shown in Table 4. The maximum temperature difference/mean temperature of the bottom surface squared ($\Delta T\left(X\right)/T_{avg}^{2}\left(X\right)$) of the heating film in the optimization results is [6.5 × 10^−5^, 3.8 × 10^−4^], corresponding to a maximum temperature difference Δ*T* of [0.44, 3.42] °C. The equivalent density ($\rho_{avg}$) of the heating film is reported to be within the range of 1.03 × 10^3^ to 1.16 × 10^3^ kg/m^3^. In order to verify the effectiveness of the optimization results, this paper uses finite element simulation experiments to verify the Pareto frontier solution set, as shown in Figure 12. The experimental results show that the prediction error of the neural network model for the maximum temperature difference Δ*T* and the equivalent density $\rho_{avg}$ of the heating film under different optimization design schemes is less than 5% and the solution of the optimization design is correct and effective. At the same time, according to the finite element results, the relationship between the temperature uniformity and the equivalent density of the bottom of the heating film is further analysed and it is found that it can be seen from the structure of the maximum temperature difference (corresponding to the red triangle in Figure 11) and the minimum temperature difference (corresponding to the red circle in Figure 11) that the lowest temperature on the bottom surface of the heating film is at the diagonal position of the “Z”-shaped heating wire, which improves the in-plane temperature of the bottom surface material. Thermal conductivity and reducing the heating wire gap can effectively increase the temperature at the diagonal position but subsequently lead to a significant increase in the equivalent density of the heating film.

**Figure 13:**
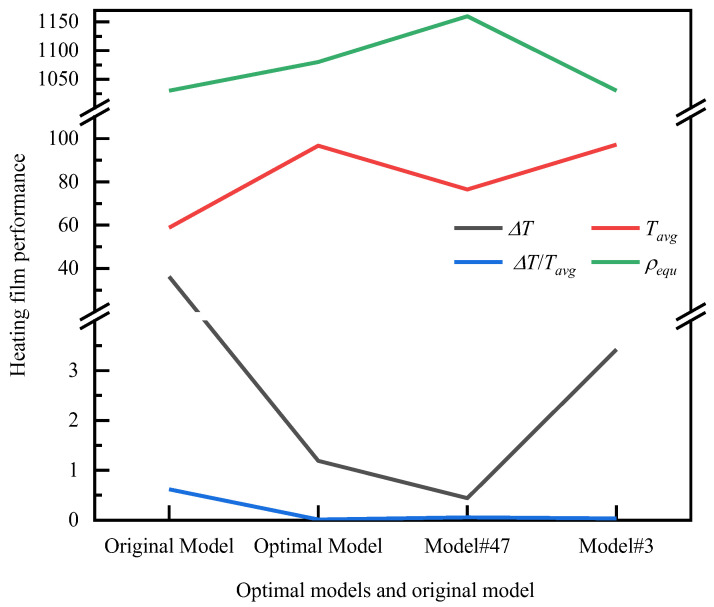
Performance comparison of the optimal models and the original model.

In considering the heating film as an aerospace thermal control unit, it is evident that both the uniformity of heating and the lightweight of the overall structure are indispensable. In order to reconcile the apparent contradiction between the two, the study selected the design scheme depicted in Table 4. As illustrated in Figure 11 and Figure 13, this solution is situated in the central region of the Pareto front solution set (represented by a red, five-pointed star in Figure 11), exhibiting a maximum temperature difference/the square of average temperature of 1.27 × 10^−4^, a maximum temperature difference of 1.188 °C, a bottom surface average temperature of 96.72 °C, and an equivalent density of 1.08 × 10^3^ kg/m^3^. A comparison of the original heating film solution with the modified version indicates that the bottom surface temperature is effectively increased under the same power and the maximum temperature difference is reduced by 35.1 °C. The temperature difference optimization effect is 30.5 times greater, and the heating efficiency and uniformity of the heater have been significantly enhanced. This advantage is reflected in the minimal increase in the equivalent density of the heating film, which is only 3.9%.

## 6. Conclusions

In response to the contradiction among the heating efficiency, uniformity, and lightweighting of anisotropic substrate heating films, which fail to meet the new thermal control requirements in aerospace, this study proposes a heating film optimization design method based on the PSO-BP neural network surrogate model. This method employs PSO to optimize the initial weights and thresholds of the BP neural network and utilizes the BP neural network to establish the mapping relationship between nine design parameters of the heating film and the uniformity of the bottom surface temperature. The results indicate that the surrogate model developed in this study effectively approximates and rapidly computes the thermal analysis of the heating film. Compared to the original design, the selected optimal design scheme significantly improves the uniformity of the temperature at the bottom of the heating film, meeting the requirement for lightweighting. The combination of neural network models and intelligent optimization algorithms not only provides an effective method for solving multi-objective optimization problems but also paves the way for interdisciplinary applications of artificial intelligence. In the future, AI-assisted design methods will not only accelerate forward design processes but also find important applications in inverse problem-based targeted design fields.

## Figures and Tables

**Table 1:** Metal heating wire material properties.

#	Metal	Thermal Conductivity (W/(m·K))	Density (kg/m³)
1	Au	315	19,320
2	Ag	429	10,490
3	Cu	398	8960
4	Al	237	2700

**Table 2:** Design parameters and interval of heating film.

Variables	Lower Limit	Upper Limit	Variables	Lower Limit	Upper Limit
*d* (mm)	0.1	0.2	*w*_1_ (%)	0	20
*l*_1_ (mm)	2	4	*w*_2_ (%)	0	20
*l*_2_ (mm)	5	10	*k* (W/(m·K))	200	500
*h* (mm)	2	4	*ρ* (kg/m^3^)	2000	20,000
*t* (mm)	0.001	0.01			

**Table 3:** Pareto front obtained by NSGA-II solution.

#	Decision Variable								Objective Function Value	
	*d* (mm)	*l*_1_ (mm)	*l*_2_ (mm)	*h* (mm)	*t* (mm)	*w*_1_ (%)	*w*_2_ (%)	Metal Number	Δ*T*/*T*^2^*_avg_*	*ρ_equ_* (kg/m^3^)
1	0.101	2.001	9.552	2.001	0.009	0.006	19.534	1	6.74 × 10^−5^	1.16 × 10^3^
2	0.101	2.002	7.655	2.002	0.003	0.011	0.272	4	3.25 × 10^−4^	1.03 × 10^3^
3	0.100	2.000	5.036	2.000	0.001	0.000	0.009	4	3.80 × 10^−4^	1.03 × 10^3^
4	0.101	2.001	5.021	2.001	0.008	0.006	9.643	4	1.09 × 10^−4^	1.09 × 10^3^
5	0.100	2.000	5.009	2.001	0.009	0.003	11.873	4	1.04 × 10^−4^	1.11 × 10^3^
6	0.103	2.001	5.047	2.001	0.001	0.006	14.588	1	9.63 × 10^−5^	1.12 × 10^3^
7	0.101	2.001	6.162	2.002	0.006	0.006	3.795	4	1.70 × 10^−4^	1.05 × 10^3^
8	0.100	2.001	5.016	2.001	0.009	0.003	7.682	4	1.13 × 10^−4^	1.08 × 10^3^
9	0.101	2.001	5.023	2.001	0.007	0.003	8.205	4	1.13 × 10^−4^	1.08 × 10^3^
10	0.101	2.001	5.066	2.001	0.009	0.006	7.098	4	1.17 × 10^−4^	1.08 × 10^3^
…				…						…
47	0.101	2.000	9.428	2.000	0.010	0.003	19.875	1	6.50 × 10^−5^	1.16 × 10^3^
…				…						…
70	0.103	2.001	5.047	2.001	0.001	0.006	14.590	1	9.63 × 10^−5^	1.12 × 10^3^

**Table 4:** Comparison of the parameters of the optimal models and the original model.

Variables	Optimal Model	Model #47	Model #3	Original Model
*d* (mm)	0.101	0.101	0.100	0.15
*l*_1_ (mm)	2.001	2.000	2.000	3.000
*l*_2_ (mm)	5.066	9.428	5.036	8.000
*h* (mm)	2.001	2.000	2.000	3.00
*t* (mm)	0.009	0.010	0.001	0.005
*w*_1_ (%)	0.006	0.003	0.000	PDMS
*w*_2_ (%)	7.098	19.875	0.009	PDMS
Wire Metal	Al	Cu	Al	Cu
Δ*T*	1.188	0.440	3.424	36.280
*T_avg_*	96.722	76.489	97.205	58.841
Δ*T*/*T*^2^*_avg_*	1.27 × 10^−4^	7.52 × 10^−5^	3.62 × 10^−4^	1.05 × 10^−2^
*ρ_equ_* (kg/m^3^)	1.08 × 10^3^	1.16 × 10^3^	1.03 × 10^3^	1.03 × 10^3^

## Data Availability

Since the data in this article are related to subsequent research, the data presented in this study are available on request from the corresponding author.
